# Dose-dependent doxycycline local drug delivery using T-PRF: preliminary in vitro study

**DOI:** 10.1038/s41598-026-53200-4

**Published:** 2026-05-21

**Authors:** Fatmanur Ezgi Doğan, Esra Ateş Yildirim, Fatma Avcioğlu, Selma Erdoğan Düzcü

**Affiliations:** 1 Department of Periodontology, Izzet Baysal Oral and Dental Health Center, Bolu, Turkey; 2 Department of Periodontology Faculty of Dentistry, Bolu Abant İzzet Baysal University, 14300 Bolu, Turkey; 3 Department of Microbiology, Faculty of Medical, Bolu Abant İzzet Baysal University, Bolu, Turkey; 4 Department of Pathology, Faculty of Medical, Bolu Abant İzzet Baysal University, Bolu, Turkey

**Keywords:** Doxycycline, Local drug delivery system, T-PRF, Antimicrobial activity, Drug release, Diseases, Drug discovery, Medical research, Microbiology

## Abstract

T-PRF is an autologous platelet concentrate widely used in medicine and dentistry. Its potential as a local drug delivery system remains an area of growing interest. This study aimed to evaluate the characteristics and performance of T-PRF membranes loaded with different doses of doxycycline (0.5 mL, 1 mL, and 2 mL). T-PRF membranes were prepared from 15 healthy individuals without bleeding disorders. Each membrane was injected with one of the three doxycycline doses, and outcomes were compared with non–drug-loaded controls. Fibrin network patterns, antibacterial activity against *Staphylococcus aureus* and *Pseudomonas aeruginosa*, doxycycline release over time, and membrane degradation rates were assessed using light microscopy and standard microbiological methods. In this preliminary in vitro study, doxycycline-loaded T-PRF membranes exhibited lower degradation rates than unloaded controls, with the 2 mL group showing the slowest degradation (*p* ≤ 0.001), suggesting that higher drug loading may enhance membrane stability. Fibrin network scores were higher in all drug-loaded groups (*p* ≤ 0.001), indicating a denser matrix that could support sustained drug retention. All membranes demonstrated antibacterial activity against S. aureus, whereas no activity was observed against P. aeruginosa, highlighting the selective antimicrobial potential of doxycycline-loaded T-PRF membranes. Among the tested groups, the 2 mL dose produced the largest inhibition zone, reflecting the most pronounced antibacterial effect within the studied range. While these results are encouraging, they should be interpreted as preliminary observations given the in vitro design, the use of the disk diffusion method, and the semi-quantitative nature of the release data. Overall, these in vitro findings indicate that doxycycline-loaded T-PRF membranes provide selective antibacterial activity and enhanced membrane stability, suggesting that autologous T-PRF may be a promising platform for local antimicrobial delivery. Further studies are needed to confirm their effectiveness and explore controlled release under more comprehensive conditions.

## Introduction

Periodontal disease is a chronic inflammatory condition caused by an imbalance between the host and microbial biofilm on tooth surfaces, with disease severity influenced by host responses and environmental factors^1,2^. Antibiotics are commonly used to treat infections caused by gram-positive and gram-negative bacteria, either systemically or locally^3^. Systemic administration has limitations, including insufficient tissue concentrations, systemic side effects, and suboptimal drug release over time. Local delivery systems have been developed to overcome these issues, providing higher and sustained drug concentrations in the periodontal pocket without systemic effects^4^.

Doxycycline is a tetracycline-class antibiotic that has long been used in periodontal therapy, achieving concentrations in gingival crevicular fluid that exceed serum levels by 2–10 times and thereby helping to prevent gingival tissue destruction. In addition to its antibacterial action, doxycycline has additional effects such as anticollagenolytic action and inhibition of bone destruction. Recently, doxycycline has been found to be important for both cementum and dentin, suggesting that it may serve as a substrate for the accumulation and subsequent slow release of doxycycline on root surfaces^5^.

Platelet-rich fibrin (PRF) is an autologous biomaterial widely used to promote soft and hard tissue regeneration. The original leukocyte-PRF (L-PRF), introduced by Choukroun et al., is prepared by centrifuging anticoagulant-free blood^6^. To overcome potential silica contamination from glass tubes, Titanium-PRF (T-PRF) was developed, providing a denser and more stable fibrin matrix with improved structural integrity. T-PRF exhibits a longer half-life, self-degrades over time, and avoids foreign-body reactions, making it a promising scaffold for local drug delivery^7^.

Staphylococcus aureus and Pseudomonas aeruginosa are clinically relevant pathogens associated with oral and periodontal infections^8–10^. P. aeruginosa, which can also be detected in peri-implant diseases and exhibits high antimicrobial resistance, is particularly important for evaluating the antibacterial performance of biomaterials. Therefore, assessing activity against these species provides a broader and clinically meaningful antimicrobial profile.

The use of local drugs as an alternative to systemic drugs in periodontal treatments has been increasing worldwide in recent years. Local drug systems are used as an adjunct to periodontal treatments by placing them into the periodontal pocket through a carrier. There are many clinical studies^11–13^. Local drug therapies have a longer release time compared to systemic drugs and can easily reach the desired concentration in the pocket. However, the disadvantages of local drug delivery systems include the fact that most of them have to be removed again with a second surgical procedure and that foreign body reactions may develop because they are not autologous. The use of T-PRF as a scaffold with a 3D matrix structure is considered a good alternative for drug delivery systems as it can self-degrade in a given time without causing any allergic/inflammatory condition in the body^14^. T-PRF has a longer half-life among other PRF types, making it more prominent as a drug carrier^7^. Although platelet-rich fibrin (PRF) has been widely studied as a regenerative biomaterial, there is limited evidence regarding its effectiveness as a local drug delivery system, particularly in relation to doxycycline loading and the impact of different doses on release characteristics and antimicrobial performance.

Although PRF and T-PRF have been extensively studied as regenerative biomaterials, evidence regarding their use as local drug delivery systems is limited. Specifically, there is a lack of data on the effect of varying doxycycline loading volumes on membrane integrity, release kinetics, and antibacterial activity. Existing studies generally evaluate a single antibiotic concentration and do not compare multiple doses to unloaded controls, leaving dose-dependent effects unaddressed.

This study aimed to evaluate doxycycline-loaded T-PRF membranes and to determine whether increasing doxycycline volumes (0.5, 1, and 2 mL) enhance drug release and antibacterial activity without compromising fibrin structure. As a preliminary in vitro investigation, it was designed to explore the suitability of T-PRF as an autologous local drug-delivery platform**.**

## Materials and methods

Our study included systemically healthy patients who were referred to Bolu Abant İzzet Baysal University Faculty of Dentistry, Department of Periodontology and who were not taking medications that affect the natural coagulation process. Patients were informed about the study and informed consent forms stating that their participation was voluntary were obtained from each patient. The study protocol was carried out according to the principles described in the Declaration of Helsinki, including all changes and revisions. The study was approved by the Bolu Abant Izzet Baysal University Local Ethics Committee (protocol no: 2023/246).

### Sample size

The sample size was calculated using the G*Power 3.1.9.2 program, assuming a Type I error of 0.05, a power of 80%, and an effect size of 0.5 based on a comparable study^15^. To account for potential data loss and experimental variability, the minimum required sample size was increased by approximately 25%, yielding a target of 15 participants. Consequently, the study was conducted with 15 systemically healthy individuals aged 22–63 years, from whom T-PRF membranes were prepared for each of the four groups (0.5 mL, 1 mL, 2 mL doxycycline, and control). Although all samples originated from the same donor pool, each membrane was processed, handled, and analyzed as an independent experimental unit under standardized laboratory conditions. Therefore, the groups were considered statistically independent for comparative analyses.

### Study design

The study was designed as an in vitro experimental investigation to evaluate the effects of different doxycycline loading volumes on the structural and functional characteristics of titanium-prepared platelet-rich fibrin (T-PRF) membranes. T-PRF membranes were obtained from venous blood samples collected from participants, and for each individual, the four membranes were randomly assigned to experimental groups (0.5 mL, 1 mL, 2 mL doxycycline, and an unloaded control) using a pre-prepared randomization table. The unloaded membranes served as the control group to provide baseline measurements of fibrin network structure, degradation behavior, and inherent antimicrobial activity, allowing direct comparison with doxycycline-loaded membranes.

Following preparation, all membranes were subjected to systematic analyses to determine fibrin network patterns, antimicrobial activity, drug-release profiles, degradation behavior, and percentage of degradation. This approach ensured unbiased allocation and allowed each membrane to serve as an independent experimental unit, enabling a reliable comparative evaluation of dose-dependent changes in membrane structure and antibacterial performance.

#### Study groups

- Group 1: Untreated T-PRF membranes (n = 15)
- Group 2: 0.5 mL doxycycline-injected T-PRF membranes (1.5 mg doxycycline) (n = 15)
- Group 3: 1 mL doxycycline-injected T-PRF membranes (3 mg doxycycline) (n = 15)
- Group 4: 2 mL doxycycline-injected T-PRF membranes (6 mg doxycycline) (n = 15)

#### Inclusion criteria

- Age between 18–65 years
- Systemically healthy
- Normal platelet counts (150,000–450,000/µL)
- Not taking any regular medication
- No conditions affecting T-PRF structure, such as platelet dysfunction

#### Exclusion criteria

- Systemic disorders or continuous medication use
- Tobacco smokers
- Bleeding disorders or platelet dysfunction
- Alcohol or substance addiction
- Pregnant or lactating women
- Systemic inflammatory conditions
- Failure to provide informed consent

### Platelet and complete blood count

Current platelet and complete blood count values were obtained from patients who agreed to participate in the study and met our inclusion criteria before any procedure was performed.

### Blood collection and T-PRF membrane preparation

In accordance with the antisepsis rules in our clinic, 40 mL blood samples were collected from the antecubital vein of each patient by a specialist using a 50 mL syringe and the blood in the syringe was divided into 10 mL titanium tubes. This procedure was repeated after 2 weeks. In total, 8 tubes of blood were obtained. For centrifugation, the titanium tubes were placed in the IntraSpin™ (Intra-Lock International, USA) table-top centrifuge, the original FDA-approved L-PRF™ centrifuge on the market, facing each other to ensure equilibrium. To obtain T-PRF, the IntraSpin™ was set to 12 min at a centrifugal force of 2700 rpm. After centrifugation, the fibrin layer in the middle of the 3 layers formed in the titanium tube was removed with the help of a press and the red layer with dense erythrocytes in the bottom layer was scraped off from the yellow fibrin layer. PRF box (Xpression™ Box Kit) was used to turn the yellow fibrin layer into a membrane. Magnetic plates with a height of 1 mm were used to ensure that the membranes had a standardized thickness. The T-PRF samples were placed on the lid weight of the PRF box for 2 min until they reached a thickness of 1 mm. After the fibrin layer was kept in the PRF box for 2 min, T-PRF membranes were obtained. Loading Doxycycline on T-PRF membranes.

**Fig. 1:**
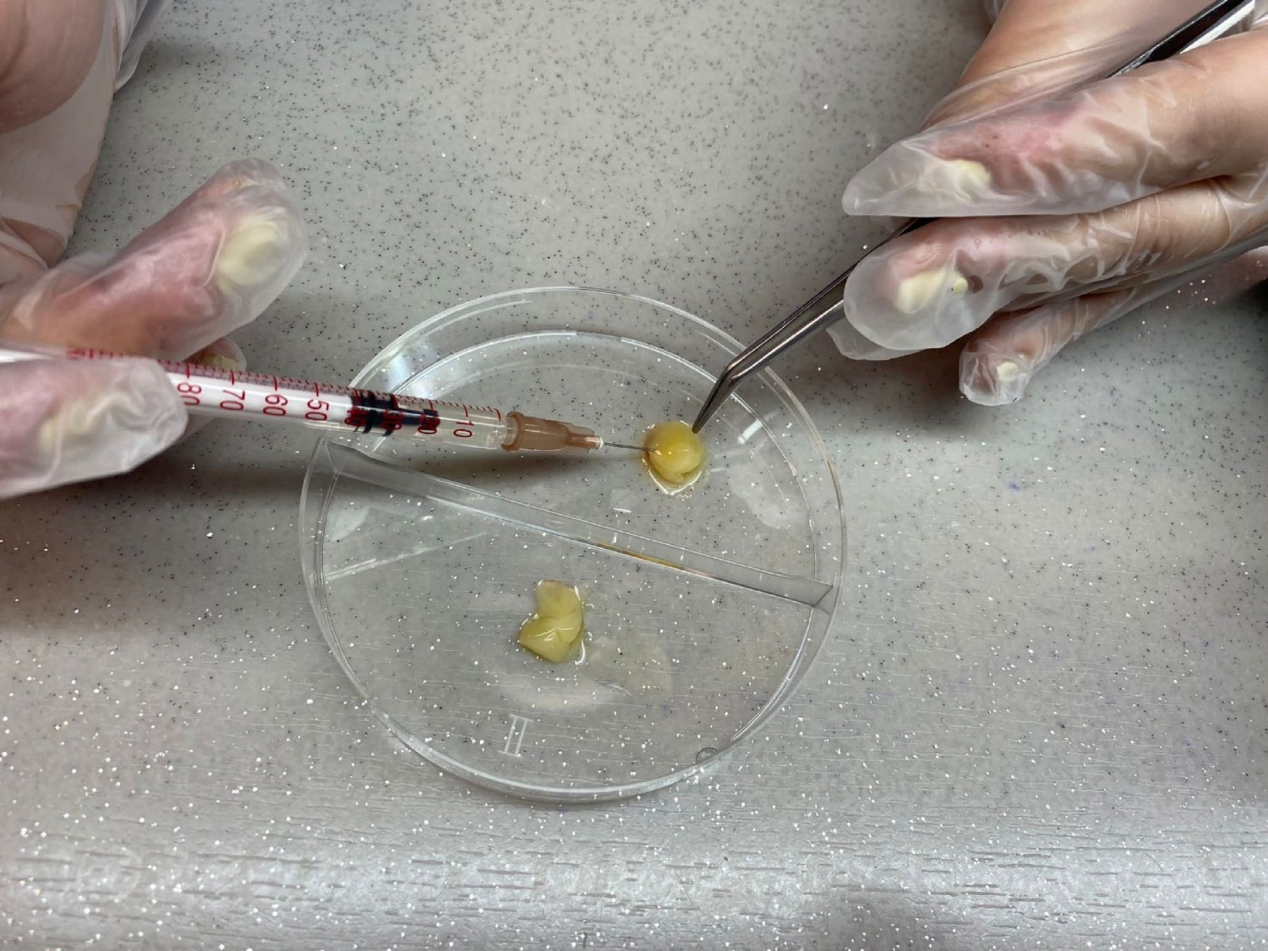
Doxycycline loading into T-PRF membrane.

Of the 8 T-PRF membrane samples obtained from individuals, 2 of them were not loaded with drugs to be used in the control group. Two of the membranes were injected with 0.5 mL Doxycycline hiklat solution at a concentration of 3 mg/mL, 2 of the membranes were injected with 1 mL Doxycycline solution at a concentration of 3 mg/mL using the same technique, and the other 2 membranes were injected with 2 mL Doxycycline solution at the same concentration (Fig. 1). To ensure procedural standardization, all doxycycline applications were performed by a single experienced periodontist. Each T-PRF membrane was positioned in a sterile Petri dish, and the doxycycline solution was carefully injected into the central region of the membrane using a 1-mL insulin syringe equipped with a 27-gauge needle. This standardized approach was adopted to maintain consistency in membrane handling, injection depth, and intramembranous distribution of the solution. The selected antibiotic doses were based on established criteria in local drug-delivery research, with the highest dose determined according to the saturation capacity of the T-PRF material, while the remaining doses were chosen by referencing comparable studies to maintain clinically relevant concentrations and allow dose-dependent evaluation^16^.

### Degradation of T-PRF membranes

Membrane weights were measured in grams using a precision balance (Sartorius CP225D). To evaluate degradation, membranes were placed on an orbital shaker (Unimax 1010) and shaken continuously for 1 week in 7.4% PBS. After 1 week, membranes were washed with distilled water, dried, and reweighed. Percentage of degradation was calculated as: % degradation was calculated as (initial weight-final weight)/initial weight × 100^17^.

### Histological examination

T-PRF membranes were histologically examined using the cell block cytology method. Membranes were transferred to cassettes and fixed in formalin solution for 24 h. After 24 h, the cassettes were dehydrated with various concentrations of formalin, alcohol and xylene. The tissue was embedded in Leucher blocks using paraffin for easy cutting and examination. Sections of 3 µm thickness were taken from the embedded blocks using a microtome. The paraffin in the blocks was removed and the tissue was stained with hematoxylin–eosin. The sections were evaluated by the pathologist under various magnifications under a LEICA DM 2000 LED light microscope.

The T-PRF clot was analyzed microscopically using the blood element adhesion index (BEAI) on hematoxylin–eosin stained slides.

- Score 0: Absence of fibrin mesh
- Score 1: Rarely dispersed fibrin network
- Score 2: Thin fibrin mesh that weakly interweaves with each other
- Score 3: Dense fibrin network with abundant interweaving
- Score 4: Very dense fibrin network with abundant interweaving

Hematoxylin–eosin stained sections were photographed at various magnifications using the Infinity 3 Analyze Release 6.5 imaging system.

### Investigation of antibacterial activity of T-PRF membranes

The prepared Doxycycline-loaded T-PRF membrane samples were divided transversely into thirds and the 2/3 part was investigated for antibacterial activity. Membranes were impregnated on blank disks (Schleicher & Schül, Nr. 2668, Germany) under sterile conditions. Disk diffusion technique was used to investigate antibacterial activities. P. aeruginosa ATCC 27,853 and S. aureus ATCC 29,213 bacteria were used as standard strains. For the preparation of bacterial stock culture, the strains were kept in 10 mL of liquid medium (OXOID) for 24 h at 37 °C oven. Then, the bacteria taken from the liquid medium were inoculated onto the solid medium, Sheep Blood agar, at a rate of 50 μL 0.5 × 108 CFU/mL. Without wasting time, disks containing Doxycycline-loaded T-PRF samples were placed on the inoculated solid medium. At the same time, penicillin (P) for S. aureus and amikacin (AK) for P. aeruginosa were used as controls. The media were incubated at 37 °C in an oven for 24 h. The next day, the inhibition zone diameters (IZD, mm) around the materials were measured relative to the clean zone diameter.

### Investigation of drug release of T-PRF membranes

Doxycycline release from the T-PRF membranes was evaluated over a 72-h period. Following preparation, each doxycycline-loaded T-PRF membrane was immediately transferred into 20 mL of PBS and incubated at 37 °C. UV–Vis measurements (Specord® 210 Plus) performed at 1, 2, 3, 4, 5, 20, 30, 48, and 72 h demonstrated a progressive increase in doxycycline concentration in the release medium. Quantification was conducted using a pre-established calibration curve, and all measurements were performed in triplicate. Cumulative release values were calculated based on the amount of doxycycline detected in PBS at each time point. Because the total amount of drug retained within the membrane was not directly measured, these values represent cumulative release relative to the detected concentration rather than absolute release efficiency. Overall, doxycycline levels continued to rise throughout the 72-h testing period, indicating that T-PRF was capable of releasing measurable amounts of drug under the in vitro conditions applied.

### Statistical analysis

All statistical analyses were performed using IBM SPSS Statistics version 25 (IBM Corp., Armonk, NY, USA). The normality of continuous variables was assessed using the Shapiro–Wilk test to determine the appropriate statistical approach. Descriptive data are presented as mean ± standard deviation (SD). For the degradation dataset, values that did not meet normality were log-transformed, and one-way ANOVA was applied; geometric means are reported accordingly. Age differences between female and male participants did not meet the normality assumption and were analyzed using the Mann–Whitney U test. Categorical variables were analyzed using the Chi-Square test. For inhibition zone diameter measurements, one-way ANOVA was performed, and since the assumption of homogeneity of variances was satisfied, Tukey’s post hoc test was applied for multiple comparisons. A *p*-value < 0.05 was considered statistically significant.

## Results

### Demographic and complete blood count data

A total of 15 patients (8 females, 7 males) aged 22–63 years were included in the study. Mean age did not differ significantly between males and females (*p* = 0.422) (Table 1). Complete blood count values for all participants were within normal physiological limits (Table 2).

**Table 1:** Demographic information of individuals.

	Age (Mean ± Sd)	Median	Min	Max	*p*-value
Female	35.625 ± 12.20	31.5	22	55	0.422
Male	44.142 ± 14.96	48	23	63	
Total	39.6 ± 13.77	34	22	63	

Descriptive statistics are presented as mean ± standard deviation (SD). Normality of the data was assessed using the Shapiro–Wilk test, and all variables were found to be normally distributed (*p* > 0.05)."Man Whitney U Test.

**Table 2:** Blood parameters of individuals.

	Mean(± Sd)	N
WBC	6.621 ± 1.4	15
RBC	4.816 ± 0.542	15
HCT	40.893 ± 2.782	15
MCV	85.6067 ± 8.813	15
PDW	15.426 ± 9.946	15
MPV	9.74 ± 1.33	15
PLT	283.133 ± 63.559	15

Descriptive statistics are presented as mean ± standard deviation (SD). Normality of the data was assessed using the Shapiro–Wilk test (WBC: Leukocyte count, RBC: Erythrocyte count, PLT: Platelet count, PDW: Platelet distribution width, MPV: Platelet volume, MCV: Average erythrocyte volume, HTC: Hematocrit).

### Chemical degradation

The degradation percentages of drug-loaded T-PRF membranes were statistically significantly lower compared to the control group (*p* < 0.05) (Table 3).

**Table 3:** Degradation data of T-PRF membranes.

Groups	Control group	0.5 ml doxycyline	1 ml doxycycline	2 ml doxycycline
N	14	15	15	15
Mean ± (LCI-UCI)	7.13 ± (3.71–13.68)ª	2.68(1.62–4.45)ᵇ	2.43(1.13–5.23)ᵇ	2.55(1.63–4.00)ᵇ
*p*-value	< 0.001*			

*Means ± SD; values within each row not sharing a common superscript are significantly different (*P* < 0.05) as determined by Least Significant Differences. Intergroup degradation data; values are geometric means (− 1 SD, + 1 SD) with statistical analysis performed on log values. One-wayANOVA test with post-hoc test Tukey correction was used. N: Number of individuals, LCI: Lower Confidence Interval, UCI: Upper Confidence Interval.

### Histological examination

**Fig. 2:**
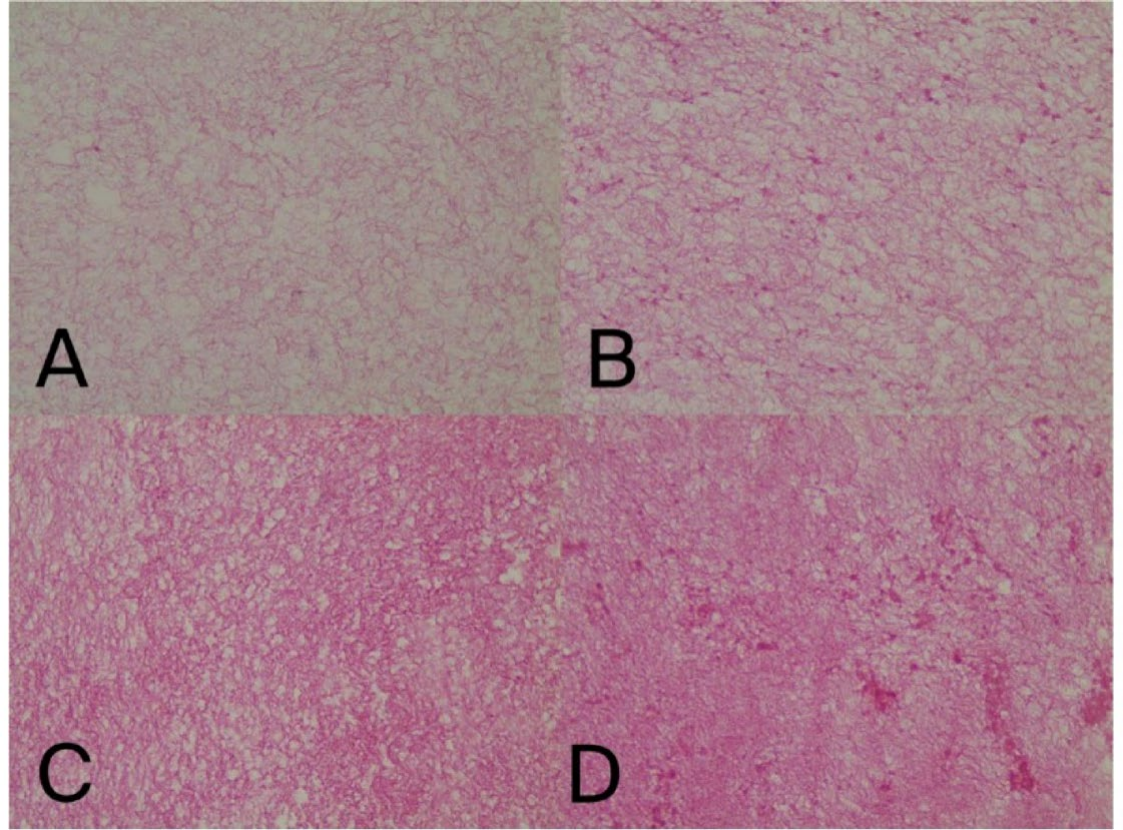
Image of T-PRF membrane by light microscopy (**A** Light microscopy image of T-PRF membrane without drug, ×400 magnification, **B** T-PRF membrane with 0.5 mL doxycycline, ×400 magnification, **C** T-PRF membrane with 1 mL doxycycline, ×400 magnification, **D** T-PRF membrane with 2 mL doxycycline, ×400 magnification).

The membranes in the test group had the lowest value of 2 and the highest value of 3 (Table 4). 2 mL drug-loaded membranes showed higher fibrin network pattern scores compared to 1 mL and 0.5 mL drug-loaded test groups (Fig. 2). This difference between the test groups was significant (*p* ≤ 0.05) (Table 5).

**Table 4:** Distribution of fibrin network pattern scores according to the number of individuals.

Fibrin network pattern score	Control group	0.5 ml doxycycline	1 ml doxycycline	2 ml doxycycline	Total	p-value
2	13	13	8	4	38	≤ 0.001*
3	2	2	7	11	22	
Total	15	15	15	15	60	

*Means ± SD; values within each row not sharing a common superscript are significantly different (*P* < 0.05) as determined by Least Significant Differences. Chi-square test.

**Table 5:** Average fibrin network pattern scores of individuals.

	Mean ± Sd	N
Control group	2 ± 0.53	15
0.5 ml doxycycline	2.13 ± 0.35	15
1 ml doxycycline	2.46 ± 0.51	15
2 ml doxycycline	2.73 ± 0.45	15
Total	2.33 ± 0.54	15

Descriptive statistics are presented as mean ± standard deviation (SD). Normality of the data was assessed using the Shapiro–Wilk test.

### Antibacterial activity

**Fig. 3:**
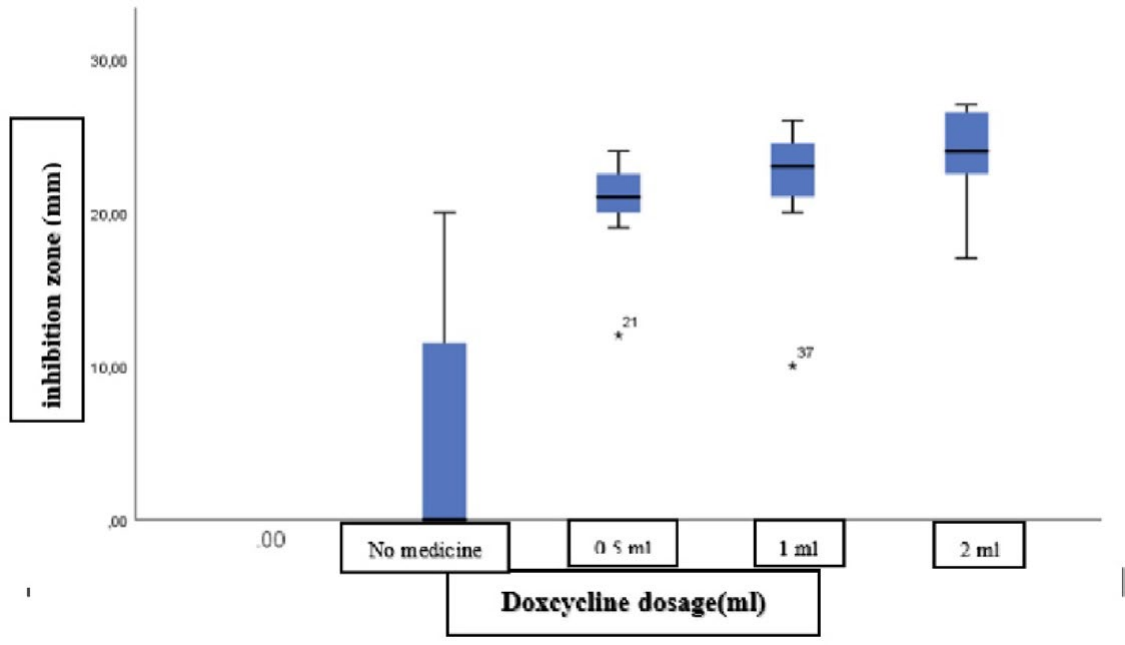
Plot of inhibition zone diamaters (mm) developed against S. aureus.

The antibacterial activity of the membranes was investigated against P. aeruginosa and S. aureus during 2 days of incubation. At the end of two days, no antibacterial activity was observed against P. aeruginosa in all groups. Antibacterial activity against S. aureus showed significant bacterial inhibition with an average IZD of 6.2 ± 0.11 mm in the control group, 21.33 ± 0.09 mm in the 0.5 mL drug loaded group, 24.66 ± 0.13 mm in the 1 mL drug loaded group and 27.33 ± 0.11 mm in the 2 mL drug loaded group. The greatest antibacterial activity against S. aureus was observed in the 2 mL drug loaded group (*p* ≤ 0.05) (Table 6). The antibacterial activity against S. aureus increased as the loaded drug dose increased(Fig. 3).

**Table 6:** Storage of inhibition zone diameters (mm) applied to S. aureus.

	N	Mean ± Sd	p-value
Control group	15	6.2 ± 0.11ª	0.001*
0.5 ml doxycycline	15	21.33 ± 0.09ᵇ	
1 ml doxcycline	15	24.66 ± 0.13ᵇ	
2 ml doxcycline	15	27.33 ± 0.11ᵇ	

*Means ± SD; values within each row not sharing a common superscript are significantly different (*P* < 0.05) as determined by Least Significant Difference. Intergroup S. aureus inhibition zone data; One-wayANOVA test with post-hoc test Tukey correction was used.

### Drug release

**Fig. 4:**
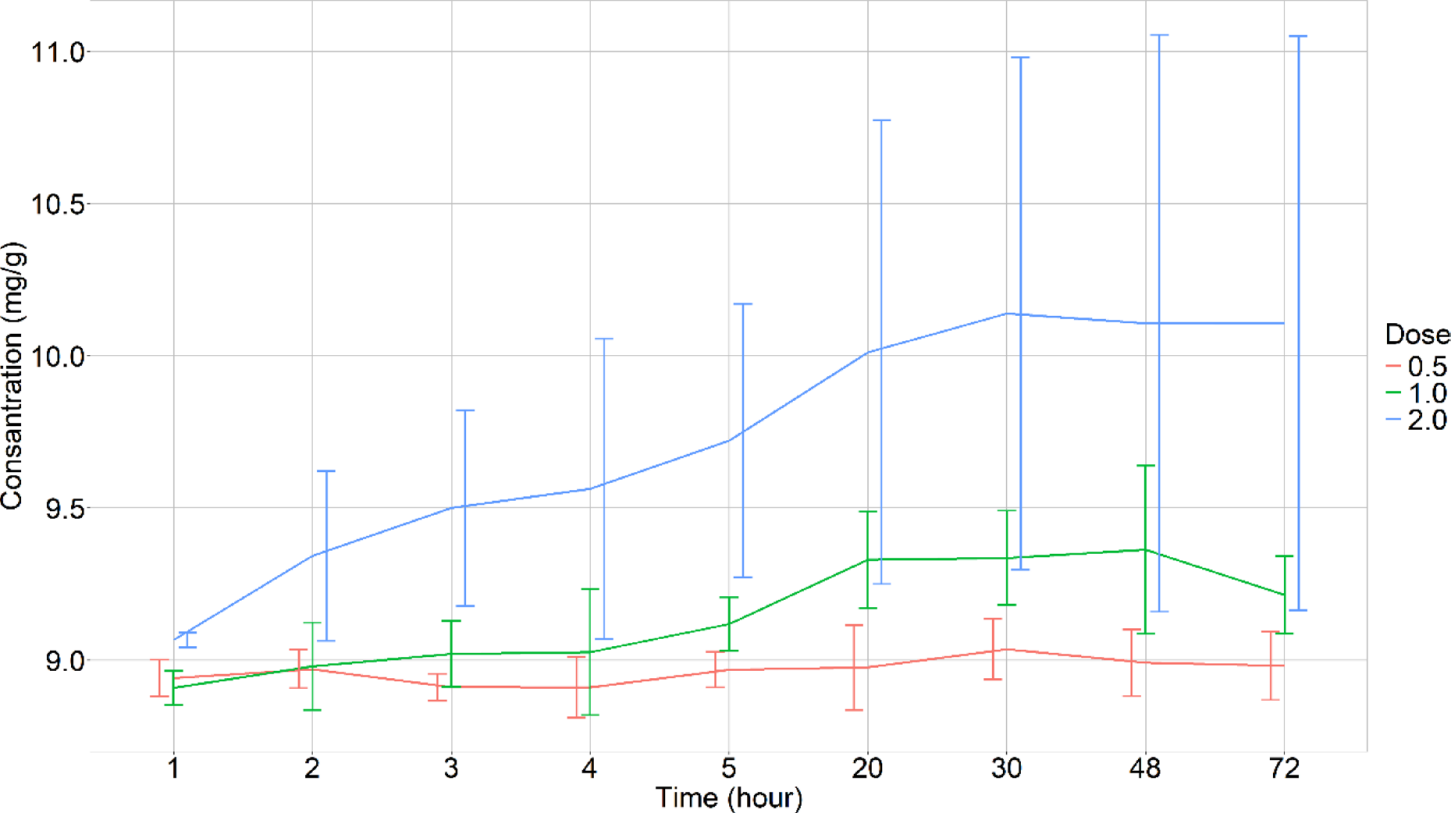
Time-dependent drug release concentrations (µg/ml) of T-PRF membranes loaded with different doses.

The doxycycline release profiles of the prepared membranes (expressed in µg/mL) are presented in Fig. 4. 0.5 mL and 2 mL drug-loaded T-PRF membranes reached maximum release capacity at 30 h and 1 mL drug-loaded TPRF membranes at 48 h. The release continued slowly until 72 h with cumulative release. Among the groups tested in this preliminary in vitro study, the 2 mL doxycycline-loaded T-PRF membrane exhibited the highest drug release. A dose-dependent increase in the concentration of drug released was observed. (*p* ≤ 0.05) Fig. 4).

## Discussion

Local drug delivery systems play a crucial role in periodontal therapy by achieving high drug concentrations at the target site while minimizing systemic exposure and the risk of bacterial resistance^18–20^. Among these, doxycycline has been widely applied directly into the periodontal pocket due to its potent antimicrobial properties. Recent efforts have focused on optimizing delivery platforms to enhance sustained release and therapeutic efficacy^21–23^. Studies have shown that carrier-based systems, such as proniosomal gels and polymeric platforms like PLGA and polyisocyanopeptides, can modulate doxycycline release kinetics, offering prolonged drug availability^24^. In a recent in vitro study, interest in autologous and carrier-free delivery systems was further highlighted, emphasizing the need for biocompatible platforms capable of sustained release^15^. However, investigations in this area remain limited..

Although T-PRF is similar to L-PRF in fibrin structure, it shows some differences. In 2020, Bhattacharya et al. compared L-PRF and T-PRF membranes in healthy individuals and found that T-PRF was more advantageous in periodontal regeneration compared to L-PRF prepared in silica glass tubes^25^. In a study conducted by Gummaluri et al. for the treatment of intraosseous defects, T-PRF was reported to be more effective in defect filling compared to L-PRF^26^. Studies support the preference of T-PRF membrane in studies because it does not contain silica glass particles, has a thicker and more prominent fibrin network, and contains a better organized fibrin and matrix structure compared to L-PRF membrane^7^.

In the literature, there are many studies in which PRF membranes are used as local drug carriers by loading antibiotics in different forms^27,28^. In the study by Alhaffar et al. in which they investigated the antibacterial activity of L-PRF membranes using different forms and doses of lincomycin, the highest antibacterial activity against S. aureus and E. faecalis bacteria was observed in the 0.5 mL ampule form of the drug-loaded L-PRF membrane, but no statistically significant difference was found in the comparison between the groups^27^.

In our study T-PRF membranes demonstrated dose-dependent antibacterial activity against S. aureus, with the largest inhibition observed in the 2 mL drug-loaded group. A small inhibitory effect was also noted in the control membranes, likely due to the intrinsic antimicrobial properties of the fibrin matrix and residual leukocytes, consistent with previous reports of modest baseline antibacterial activity in autologous fibrin matrices^29,30^. No antibacterial activity was observed against P. aeruginosa, which is consistent with its well-known intrinsic resistance to many antibiotics, including doxycycline. This finding highlights that while T-PRF membranes loaded with doxycycline are effective against susceptible pathogens such as S. aureus, they may not provide significant inhibition of intrinsically resistant species. Clinically, this suggests that doxycycline-loaded T-PRF is most suitable for targeting periodontal pathogens that are sensitive to tetracyclines, and additional or alternative antimicrobial strategies may be required to manage infections involving resistant organisms like P. aeruginosa.

In a study comparable to ours that employed the disk diffusion method, A-PRF membranes demonstrated antimicrobial activity against S. aureus, H. influenzae, and E. coli in individuals with jaw osteonecrosis who were undergoing treatment with sulbactam and ampicillin^31^. Consistent with this approach, another investigation reported that PRF and CGF exhibited significantly greater inhibition zone diameters compared with control groups, while membranes loaded with amoxicillin and metronidazole demonstrated superior antibacterial activity across all tested strains^9^. A recent study demonstrated that PRF and T-PRF membranes loaded with clindamycin or amoxicillin–metronidazole significantly inhibited Aggregatibacter actinomycetemcomitans and Porphyromonas gingivalis, highlighting the potential of antibiotic-loaded fibrin membranes as local antibacterial agents in periodontal therapy^20^. Additionally, in a separate study employing the disk diffusion technique, T-PRF supplemented with the doxycycline showed markedly greater inhibition diameters than both the collagen matrix and T-PRF alone^15^. Castro et al. found that L-PRF membrane showed antibacterial effect against periodontopathogens such as P. intermedia, F. nucleatum and A. actinomycetemcomitans^32^. In a study by Bielecki et al., similar to our study, platelet-rich gel (PRP) inhibited the growth of S. aureus, but did not show any antibacterial activity against P. aeruginosa and even induced the growth of P. aeruginosa in vitro. ^33^.

Polak et al. loaded three different antibiotics (metronidazole, clindamycin, and penicillin) at three doses (0.5 mL, 1 mL, and 2 mL) onto L-PRF membranes before centrifugation and investigated their antibacterial activities against *S. aureus* and *F. nucleatum*, as well as the physical properties of the membranes. In their study, the 1 and 2 mL drug-loaded groups could not be evaluated for antibacterial activity because centrifugation disrupted the PRF structure. In contrast, the 0.5 mL groups showed significant antibacterial activity against both bacteria for up to four days, with clindamycin- and penicillin-loaded membranes demonstrating statistically greater activity against *S. Aureus*^16^.

Current research includes studies assessing antibacterial activity against a variety of microbial species^9,34^. Based on previous findings, we selected *P. aeruginosa*, known for its strong biofilm-forming capacity and antibiotic resistance, and *S. aureus*, commonly isolated from periodontal and peri-implant sites, to ensure microbiological relevance in our model^10,16,34^.

The antibacterial activity observed in our study is consistent with previous reports highlighting the role of fibrin in modulating antibacterial effects or in other drug delivery systems^35^. The architecture of the fibrin network—including fiber density, degree of cross-linking, and pore size—can influence drug entrapment and diffusion, thereby affecting antibacterial performance^36^. These structural features likely contribute to the retention and gradual diffusion of doxycycline from T-PRF membranes, supporting previous findings that autologous fibrin scaffolds can serve as effective natural delivery platforms ^15^. Moreover, comparative studies of different delivery systems have shown that tetracycline fibers outperform doxycycline and chlorhexidine gels in clinical parameters while providing more controlled drug release^11^. In this study, antibacterial activity was assessed using the disk diffusion method, which primarily reflects diffusion-based inhibition rather than controlled release kinetics; therefore, direct correlations between antibacterial outcomes and sustained release profiles should be interpreted with caution. In a recent clinical study, adjunctive use of i-PRF and its ciprofloxacin-loaded formulation in non-surgical periodontal therapy demonstrated that the ciprofloxacin-enriched i-PRF group achieved significantly greater clinical attachment gain, probing depth reduction, and decreases in *A. actinomycetemcomitans* levels compared with the other treatment groups over a three-month period. These findings suggest that integrating an antimicrobial agent into the i-PRF matrix may potentiate its therapeutic effects by combining the regenerative capacity of platelet-derived biomolecules with targeted antibacterial activity. The enhanced outcomes observed in the antibiotic-loaded group may be attributed to prolonged local drug availability facilitated by the fibrin network, which can act as a natural reservoir and modulate drug diffusion^37^. From a biological perspective, the simultaneous delivery of growth factors and a locally sustained antimicrobial concentration may create a more favorable environment for periodontal healing by suppressing pathogenic bacterial load while supporting soft-tissue regeneration. This mechanistic interplay highlights the potential of customized, drug-enhanced autologous preparations as an advanced approach to amplifying the clinical benefits of non-surgical periodontal therapy.

In our study, the membranes in the control group exhibited significantly higher degradation rates compared with the drug-loaded membranes. There are no studies in the literature examining the degradation rate of T-PRF membranes loaded with different doses of doxycycline, but in a similar study by Sam et al., the physical properties of T-PRF membrane obtained from healthy individuals were compared with collagen membranes, and it was found that approximately 36% of the initial weight of the T-PRF membrane degraded after a one-week in vitro shaking test^38^. Ravi et al. evaluated the degradation rates of A-PRF, L-PRF and T-PRF membranes. The membranes were kept in pH 7.4 PBS solution on an orbital shaker at 50 rpm for one week. At the end of the seventh day, the degradation rate of the L-PRF membrane was the highest with 85%, the degradation rate of the A-PRF membrane was the second highest with 84% and the degradation rate of the T-PRF membrane was the lowest with 82%^17^. The differences observed in degradation behavior among the doxycycline-loaded T-PRF membranes can be explained by underlying structural and biological mechanisms. Doxycycline is known to bind to fibrin fibers and plasma proteins, which may contribute to a denser and more stabilized fibrin architecture, thereby reducing enzymatic degradation rates compared with unloaded membranes.

In our study, with reference to similar studies in the literature^38,39^, T-PRF membranes were kept in PBS solution in an orbital shaker for one week. Differences between studies may be due to reasons such as the method of obtaining PRF and individual differences.

There are no studies to date examining the fibrin network patterns of T-PRF membranes loaded with different doses of doxycycline. However, related research provides insights into factors influencing fibrin architecture. Yajamanya et al. reported that fibrin network density decreases and loose fibrin arrangements increase with advancing age in PRF membranes^40^. In a recent study evaluating the effect of different g-forces applied during the production of horizontally centrifuged H-PRF, SEM analyses demonstrated that increasing the centrifugal force resulted in thinner fibrin bundles and a markedly denser fibrin network. This finding supports the notion that the dense fibrin architecture observed in T-PRF membranes is closely influenced by centrifugation parameters and the dynamics of fibrin polymerization^41^. These findings suggest that both biological factors (e.g., age, systemic health) and technical parameters—particularly centrifugation force and protocol—significantly influence fibrin organization. Such variations in fibrin density and architecture may, in turn, affect drug-loading capacity, release kinetics, and membrane stability.

In a study evaluating CGF and PRF as local drug-carrier systems, membranes were treated with clindamycin and ampicillin–clavulanic acid prior to centrifugation; however, high concentrations of clindamycin led to noticeable membrane degradation^9^. Similarly, another investigation incorporated linezolid, vancomycin, and gentamicin into PRF membranes before centrifugation and reported that vancomycin inhibited proper membrane formation^10^. Considering the risk of impaired membrane integrity and the potential inability to achieve the desired drug concentration with pre-centrifugation loading, doxycycline was administered into the membranes post-centrifugation in the present study. In a study comparable to ours, the release characteristics of A-PRF combined with different antibiotics were evaluated over a 14-day period, and amoxicillin was found to exhibit a higher release rate than metronidazole^42^. In a study by Ercan et al., the drug release amounts of doxycycline-loaded T-PRF membrane and doxycycline-loaded collagen sponge were examined for 72 h, similar to our study. Only 25% of the doxycycline-loaded T-PRF membrane was released at 24 h, while the majority of the remaining amount continued to be released throughout the 72-h study period. Under in vivo conditions, the remaining amount of the drug is expected to be released as T-PRF degrades. Therefore, these results demonstrate the potential of T-PRF as an in vivo drug carrier^15^. In another study in which PLGA microspheres loaded with doxycycline hyclate were applied into the periodontal pocket as an adjunct to non-surgical periodontal treatment in patients with chronic periodontitis, gingival groove fluid was examined from the sites on days 2, 5, 7, 10, 15 and 20 after drug administration to determine the amount of doxycycline released into the periodontal pocket. On days 2, 5 and 7, doxycycline concentration remained constant, while a decreasing trend was observed on days 10 and 15. The developed local drug delivery system successfully demonstrated sustained release after application^43^.

A recent in vitro study demonstrated that both collagen sponges and T‑PRF membranes loaded with multiple antibiotics were capable of sustained and controlled release over 72 h as measured by UV‑Vis spectrophotometry, supporting the potential of fibrin-based matrices as effective drug carriers^44^. In line with these findings, the dose-dependent increase in doxycycline release observed in our study—particularly in the 2 mL group—may be explained by a steeper concentration gradient within the fibrin network, which enhances passive diffusion. Higher drug volumes may also saturate binding sites within the T‑PRF matrix, reducing matrix–drug interactions and allowing more drug to be released over time, further supporting the role of T‑PRF as an effective sustained-release platform. Clinically, Chandrasekar et al. reported that metronidazole-loaded PRF used as an adjunct to non-surgical periodontal therapy significantly improved bleeding on probing and gingival index, although other parameters did not show statistical significance^45^. Together, these studies suggest that while fibrin scaffolds can achieve controlled antimicrobial release in vitro, translating this into meaningful clinical outcomes depends on host response, microbial environment, and the specific drug used, highlighting the relevance of integrating mechanistic and biological perspectives when evaluating T‑PRF as a local drug delivery system.

A key limitation of this study is that the evaluation of T-PRF as a potential controlled drug-release system relied on a limited set of in vitro parameters, which does not fully capture the complexity of its drug-loading and release behavior. Although cumulative doxycycline release was quantified using a validated calibration curve, the total amount of drug actually incorporated and retained within the T-PRF membrane was not directly measured. As a result, the reported release values should be interpreted as relative indicators rather than absolute release efficiency, and the initial burst release observed at early time points may reflect the presence of loosely bound or surface-associated drug. Mechanistic analyses—such as direct assessment of doxycycline distribution within the membrane matrix, quantification of drug-entrapment efficiency, drug-retention capacity, and identification of intra-membrane diffusion gradients—were not performed. Although the drug was consistently applied through the central region of each membrane to ensure procedural standardization, this approach cannot substitute for analytical verification of distribution homogeneity or true incorporation efficiency.

Additional methodological limitations include the absence of comparison groups such as free doxycycline solutions or alternative PRF derivatives, which restricts the interpretability of the release behavior. Classical sink-condition validation was not performed, although the release medium volume was intentionally selected to minimize the risk of doxycycline saturation. Likewise, while release measurements were conducted in triplicate over a 72-h period, formal assessments of doxycycline stability in PBS and UV–Vis baseline controls were not included, which may have influenced the absolute accuracy of the release profile.

Another limitation arises from the partial standardization of the T-PRF membranes. Although membrane thickness was controlled, natural biological variability in surface area and weight may have occurred, potentially contributing to minor variations in release kinetics. The study was also performed at a single center and under short-term in vitro conditions, limiting the generalizability and clinical extrapolation of the findings. Collectively, these factors indicate that the current work should be regarded as an initial exploratory study. While the system does not demonstrate fully controlled release, the ability of T-PRF membranes to sustain doxycycline release for up to 72 h suggests potential applicability. Future research incorporating direct quantification of retained drug content, optimization of loading protocols, expanded control groups, extended release monitoring, and in vivo validation is required to more comprehensively characterize the drug-release behavior of doxycycline-loaded T-PRF membranes.

## Conclusion

In our study, the fibrin density, degradation rate, and antimicrobial activity of non–drug-loaded membranes were consistently lower than those observed in the drug-loaded groups. The T-PRF membrane supplemented with 2 mL of doxycycline demonstrated the longest release duration, the densest fibrin architecture, and the highest antibacterial activity among the evaluated conditions. These observations, however, should not be interpreted as identifying 2 mL as an effective or optimal dose; it reflects only the upper limit of the doses examined. Accordingly, the findings represent preliminary feasibility outcomes rather than evidence of an established controlled-release system. Given the inherent constraints of the in vitro experimental design, the results should be considered exploratory and interpreted with caution.

Future investigations involving broader dosing ranges, extended monitoring periods, and in vivo or ex vivo models are necessary to more fully assess the biological relevance and potential therapeutic implications of doxycycline-loaded T-PRF membranes.

## Data Availability

The datasets used and/or analysed during the current study will be made available from the corresponding author on reasonable request **.**.
